# The Development and Effectiveness of a Self-Efficacy Enhancement Program for Older Adults With MCI

**DOI:** 10.1093/geroni/igaa057.035

**Published:** 2020-12-16

**Authors:** Jungeun Lee

**Affiliations:** Cheju Halla University, Jeju, Republic of Korea

## Abstract

Purpose: This study aimed to develop a self-efficacy enhancement program and examine its effectiveness in enhancing dementia preventive behaviors by improving cognitive function, dementia knowledge, and self-efficacy, and reducing depression of older adults with mild cognitive impairment (MCI). Methods: An equivalent control group pretest-posttest design was conducted at an advanced general hospital in Seoul. Participants of older adults with MCI visiting clinics were randomly allocated to an experimental group (EG, n=16) and a control group (CG, n=16). The EG was provided with a 8-week intervention (60-minute, weekly) utilizing self-efficacy enhancement strategies and the CG received the usual care. The intervention was an integrated configuration making up of physical, cognitive, and emotional activity and followed by a 4-week maintenance during which both groups engaged in self-learning at home with a dementia preventive guidebook. Outcomes were evaluated at pretest, 1st (8th week), 2nd (10th week) and 3rd posttest(12th week). Results: There were significant differenes in cognitive functions, dementia knowledge, self-efficacy, and dementia preventive behaviors, but not in depression between two groups over the time. Regarding cognitive function’ subdomains, significant differences were observed for visuospatial/executive, attention, language, and delayed recall. Conclusions. The integrated intervention consisting of physical, cognitive, and emotional activities, not a simple merger of single intervention focused on cognitive reinforcement, was effective in improving their cognitive functions, dementia knowledge, self-efficacy, and dementia preventive behaviors. It suggests that this program can be utilized as an education program to prevent dementia for MCI in dementia support centers, public health centers, clinics, and hospitals.

